# Conditional survival after surgical resection of primary retroperitoneal tumors: a population-based study

**DOI:** 10.1186/s12935-021-01751-z

**Published:** 2021-01-20

**Authors:** Shutao Zhao, Yixuan Zhao, Shuang Liu, Chao Zhang, Xudong Wang

**Affiliations:** Department of Gastrointestinal Nutrition and Hernia Surgery, The Second Hospital of Jilin University, Changchun, 130000 China

**Keywords:** Conditional survival, Dynamic assessment, Primary retroperitoneal tumors, Prognosis

## Abstract

**Background:**

The purpose of this study was to assess conditional survival (CS) after resection of primary retroperitoneal tumors (RPTs).

**Methods:**

The data of 1594 patients with primary RPTs who underwent surgery between 2004 and 2016 were retrieved from the Surveillance Epidemiology and End Results (SEER) database. Multivariate Cox analysis was used to identify prognostic factors affecting overall survival (OS) and cancer-specific survival (CSS). CS was used to calculate the probability of survival for an additional 3 years after the patient had survived x years, according to the formulas: COS3 = OS (x + 3) /OS (x) and CCSS3 = CSS (x + 3)/CSS (x).

**Results:**

The 1-, 3-, and 5-year OS rates of all patients were 89.8, 71.8, and 60.8%, while the 1-, 3-, and 5-year CSS rates were 91.9, 77.1, and 67.8%, respectively. Age, sex, FNCLCC grade, size, multifocality, histology, and chemotherapy were independent prognostic factors for OS and CSS. Among patients who survived for 1, 3, and 5 years, the COS3 rates were 72.9, 77.9, and 79.3%, and the CCSS3 rates were 78.1, 82.7, and 85.8%, respectively. Patients with poor clinicopathological characteristics achieved greater improvements in COS3 and CCSS3 rates, and the survival gaps between OS and COS3, as well as CSS and CCSS3 were more obvious.

**Conclusion:**

Postoperative CS of RPTs was dynamic and increased over time. CS increased more significantly in patients with poor clinicopathological characteristics.

## Introduction

A retroperitoneal tumor (RPT) refers to a primary highly malignant tumor in the retroperitoneal space, including the sacral and pelvic floor spaces, which is mainly classified as a liposarcoma, leiomyosarcoma (LMS), malignant fibrous histiocytoma (MFHC), malignant peripheral schwannoma, or synovial sarcoma, among others. Notably, malignant retroperitoneal sarcomas account for about 80% of RPTs. The most common sites of secondary RPTs due to retroperitoneal metastasis include the liver, gallbladder, pancreas, spleen, kidney, gastrointestinal tract, bladder, uterus, ovary, and other parenchymal organs. The incidence of a RPT is 0.5–1.0 cases per 100,000 population. However, the incidence of RPTs has continued to significantly increase in recent years, especially in younger patients. The characteristics of RPTs include deep locations, insidious onset, and the lack of specific early serological and other diagnostic markers. Radical resection of a RPT is often difficult, especially for larger tumors that invade or squeeze the surrounding organs, and the postoperative recurrence rate is relatively high. The majority of RPTs are insensitive to radiotherapy and chemotherapy, rendering treatment rather challenging [1–8].

For malignant tumors, an accurate prognostic evaluation is essential for clinical decision-making, determining monitoring strategies, and predicting short- and long-term survival outcomes. Due to the lack of staging criteria for RPTs, soft tissue sarcomas are referenced here to assess the stage of disease and to evaluate prognosis based on the tumor grade and size, lymph node metastasis, and distant metastasis [9–11]. Previous reports have identified multifocality, age, and scope of surgery as prognostic factors [10, 12, 13], indicating that there is not yet a consensus on the prognostic factors of RPTs.

Conventionally, the survival rate is predicted based on postoperative clinicopathological characteristics at a fixed time point. In fact, because the risk of death varies over time, this approach has limitations, especially for prediction of long-term survival. Conditional survival (CS) is a concept that takes into account changes in survival risk due to extended lifespan and is used to describe dynamic survival probabilities [14–16]. Because the risk of death changes over time, CS can provide more accurate information for long-term prognosis and has been used for prognostic evaluation of tumors of the gastrointestinal tract, liver, bone marrow, oral cavity, pancreas, urinary tract, and hematopoietic system, among other locations [17–25].

Until now, no study has yet to assess CS after resection of RPTs. Therefore, the aim of the present study was to evaluate the dynamic survival of patients with RPTs by referencing the surveillance epidemiology and end results (SEER) database.

## Materials and methods

### Patient population

Data of patients with primary RPTs diagnosed between 2004 and 2016 were retrieved from the SEER database using SEER*Stat statistical software (version 8.3.6). Inclusion criteria were: (1) pathologically confirmed RPTs (ICD-O-3: 48.0), (2) no prior neoadjuvant radiation, (3) underwent surgery, (4) primary RPTs, and (5) complete follow-up and survival data. The included variables included age, sex, race, marital status, French Federation of Cancer Centers Sarcoma Group (FNCLCC) grade, size, multifocality, histology, chemoradiotherapy information, extension of resection, and survival information. If any of these variables were unknown, the data set was excluded from analysis.

### Statistical analysis

In this study, overall survival (OS) is defined as the time from the start of randomized treatment to death due to any reason. In the SEER database, cancer-specific survival (CSS) was defined as the time from the start of randomized treatment to death due to a specific disease (i.e., primary RPT in this study) [26]. Survival analysis was performed using the Kaplan–Meier method. The log-rank test was used to calculate differences in survival rates among groups. The Cox proportional hazard regression model was used to identify correlations between different variables and OS and CSS. All data analyses were conducted using R software 4.0.0 (https://cran.r-project.org/src/base/R-4/) and IBM SPSS Statistics for Windows, version 21.0. (IBM Corporation, Armonk, NY, USA). A two-tailed probability (*p*) value of < 0.05 was considered statistically significant.

CS was used to calculate the possibility of additional survival for y years after the patient had survived x years with the formula CS (y | x) = S (x + y)/S, where S is OS or CSS at a certain time point [27]. In this study, COS (x) and CCSS (x) were used instead of S(x) to calculate the number of patients who were alive in year x. Here, CS at 3 years (COS3) = OS (x + 3)/OS(x) and CCSS3 = CSS (x + 3)/CSS(x). Differences in CS among groups were calculated using the standardized differences (*d*) method [28], as *d* = (*P*2 − *P*1)/√ [*P* (1 − *P*)]. The term |*d*|< 0.1 denotes no difference in each group, whereas 0.1 ≤|*d*|< 0.3 denotes a small difference, 0.3 ≤|*d*|< 0.5 denotes a moderate difference, and |*d*|≥ 0.5 denotes a significant difference.

## Results

### Clinicopathological characteristics

**Fig. 1:**
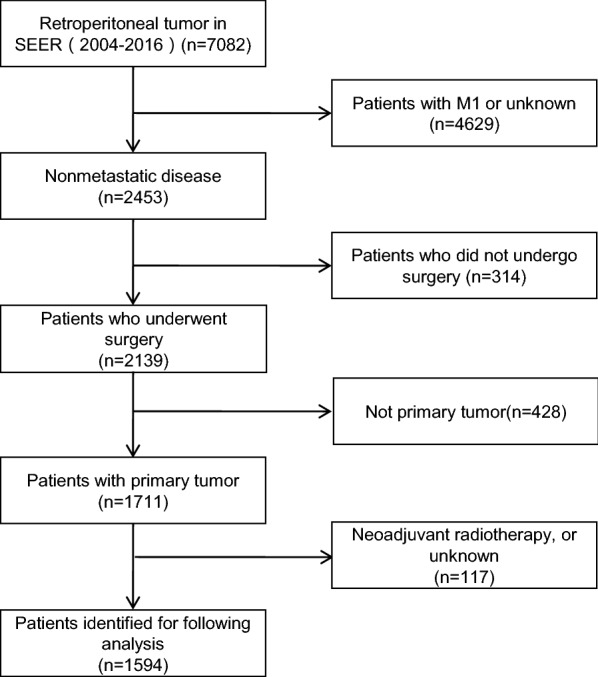
Flowchart of the selection process of included patients

The data of 1,594 patients (median age, 60 years; percent aged < 65 years, 60.8%; percent female, 54.3%) who underwent surgical resection and met the inclusion criteria for primary RPTs were included for analysis (Fig. 1). The median survival time was 42 (range, 0–155) months. The proportions of white and married patients were 79.5 and 61.4%, respectively (Additional file 1: Table S1). According to the FNCLCC grade, the highest proportions of patients had stage III disease, tumor size of ≥ 15 cm, and a single tumor (19.6, 56.5, and 86.1%, respectively). The most common pathological type was LMS (24.0%), complete resection was achieved in most patients (52.7%), and the proportions of patients receiving radiotherapy, chemotherapy, and chemoradiotherapy were low (24.7%, 13.4%, and 4.6%, respectively).

### Actual OS and CSS

**Fig. 2:**
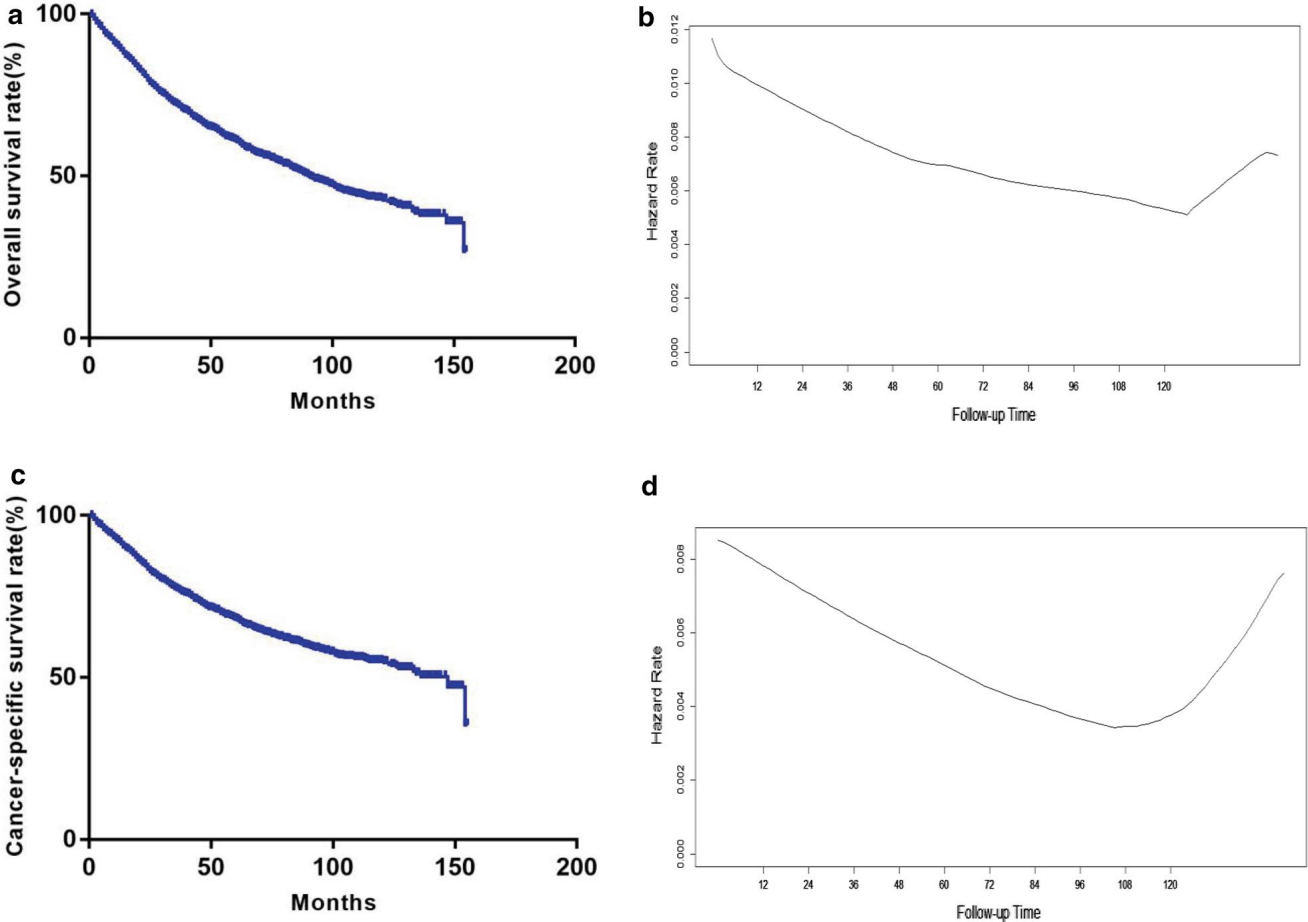
Kaplan–Meier survival curve of overall survival (**a**) and cancer-specific survival (**c**); hazard estimates of death from any reason (**b**) or primary retroperitoneal tumors (**d**) are illustrated for all patients in the cohort

At the end of the last follow-up (median follow-up, 64.0 months), a total of 693 patients (43.5%) had died, of which 523 (75.5%) succumbed to cancer-specific causes. The 1-, 3-, and 5-year OS rates were 89.8%, 71.8%, and 60.8% (Fig. 2a), while the 1-, 3-, and 5-year CSS rates were 91.9, 77.1, and 67.8%, respectively (Fig. 2c). Univariate analysis showed that age, sex, marital status, FNCLCC grade, tumor size, multifocality, histology, chemotherapy, and chemoradiotherapy were predictive of the OS and CSS rates (Additional file 2: Table S2). Furthermore, age, sex, marital status, FNCLCC grade, multifocality, histology, and chemotherapy were independent prognostic factors of OS and (excluding marital status) CSS (all, *p* < 0.05; Table 1).

**Table 1:** The multivariate analyses of factors associated with overall survival and cancer-specific survival

Variable	OS-multivariate Cox regression		CSS-multivariate Cox regression	
	HR (95% CI)	P-value	HR (95% CI)	P-value
*Age*				
< 65				
≥ 65	1.915 (1.636–2.241)	< 0.001	1.698 (1.417–2.035)	< 0.001
*Sex*				
Male				
Female	0.778 (0.663–0.913)	0.002	0.814 (0.680–0.975)	0.025
*Race*				
White				
Black				
API				
Other				
Marital status				
Married				
Unmarried	1.078 (0.863–1.347)	0.510		
Unknown	1.282 (1.069–1.538)	0.007		
*FNCLCC grade*				
I				
II	1.846 (1.130–3.016)	0.014	1.942 (1.060–3.560)	0.032
III	3.119 (2.075–4.688)	< 0.001	3.569 (2.128–5.985)	< 0.001
Unknown	1.986 (1.359–2.902)	< 0.001	2.263 (1.385–3.697)	0.001
*Size (cm)*				
< 5				
5–10	1.105 (0.719–1.697)	0.650	1.048 (0.635–1.730)	0.855
10–15	1.397 (0.905–2.155)	0.131	1.377 (0.830–2.285)	0.215
≥ 15	1.832 (1.220–2.752)	0.004	1.843 (1.147–2.961)	0.011
Unknown	2.623 (1.567–4.393)	< 0.001	2.366 (1.282–4.367)	0.006
*Multifocality*				
No				
Yes	0.588 (0.464–0.746)	< 0.001	0.111 (0.063–0.198)	< 0.001
*Histology*				
SFT				
MFHC	2.942 (1.280–6.763)	0.011	2.626 (1.061–6.497)	0.037
MPNST	1.914 (0.635–5.775)	0.249	0.727 (0.145–3.639)	0.698
LMS	1.578 (0.734–3.392)	0.242	1.496 (0.655–3.419)	0.339
DD lipo	1.404 (0.657–2.999)	0.382	1.276 (0.562–2.900)	0.560
WD lipo	0.679 (0.311–1.482)	0.331	0.435 (0.183–1.034)	0.060
Other	1.361 (0.638–2.901)	0.425	1.242 (0.548–2.813)	0.603
*Radiation*				
No				
Yes				
*Chemotherapy*				
No				
Yes	1.606 (1.320–1.953)	< 0.001	1.810 (1.464–2.238)	< 0.001
*Chemoradiotherapy*				
No				
Yes				
*Extent of resection*				
Complete				
Incomplete				
Unknown				

*SFT* solitary fibrous tumor, *MFHC* Malignant fibrous histiocytoma, *MPNST* malignant peripheral nerve sheath tumor, *LMS* leiomyosarcoma, *DD lipo* dedifferentiated liposarcoma, *WD lipo* well-differentiated liposarcoma, *FNCLCC* French National Federation of the Centers for the Fight Against Cancer, *API* Asian/Pacific Islander, *OS* overall survival, *CSS* cancer-specific survival

### COS and comparison with actual OS

**Fig. 3:**
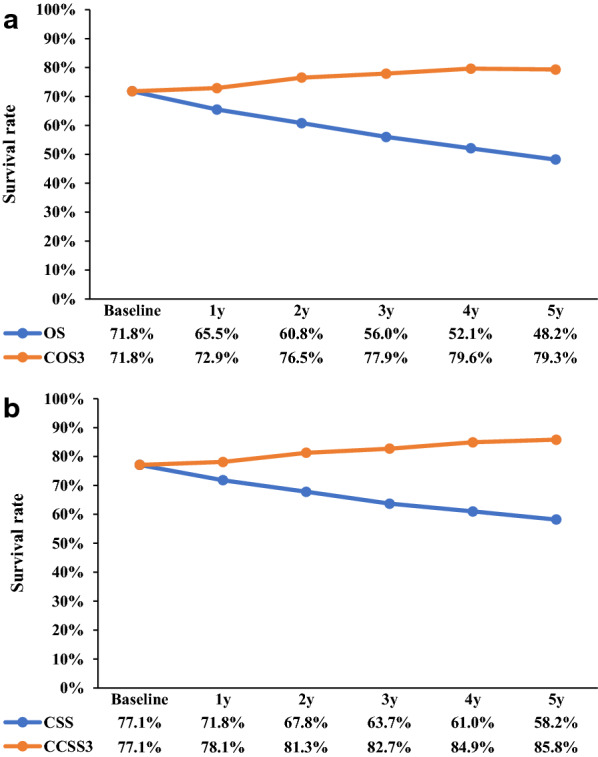
**a** Conditional overall survival relative to actual overall survival; **b** Conditional cancer-specific survival relative to actual cancer-specific survival

Analysis of the risk of disease progression over time revealed that the risk of death decreased with time after surgery, but increased after 10 years (Fig. 2b). Similarly, the risk of dying from cancer decreased over time, but increased after 9 years (Fig. 2d). The 3-year OS and COS rates are shown in Fig. 3a. The COS3 rate increased yearly after surgery, while the actual OS rate had decreased. The COS3 rate increased by 1.1 (72.9%), 6.1 (77.9%), and 7.5 (79.3%) at 1, 3, and 5 years respectively, while the corresponding actual OS rate decreased by 6.3 (65.5%), 15.8 (56.0%), and 23.6 (48.2%) at 4, 6, and 8 years, respectively. The CS rates at specific points in time are presented in Table 2. For example, among patients surviving at 1, 2, 3, and 4 years after surgery, the probability of survival at 5 years was 67.8, 76.5, 84.7, and 93.0%, respectively.

**Table 2:** The probability that patients with primary retroperitoneal tumors after surgical resection will remain alive at a specific time point given that they have already survived for a certain amount of time

Total overall survival time, y	If the patient has survived, %							
	1y	2y	3y	4y	5y	6y	7y	8y
1	100							
2	88.5	100						
3	80.0	90.3	100					
4	72.9	82.3	91.1	100				
5	67.8	76.5	84.7	93.0	100			
6	62.3	70.4	77.9	85.5	92.0	100		
7	58.0	65.5	72.5	79.6	85.6	93.1	100	
8	53.7	60.7	67.1	73.7	79.3	86.2	92.6	100

*y* year

**Fig. 4:**
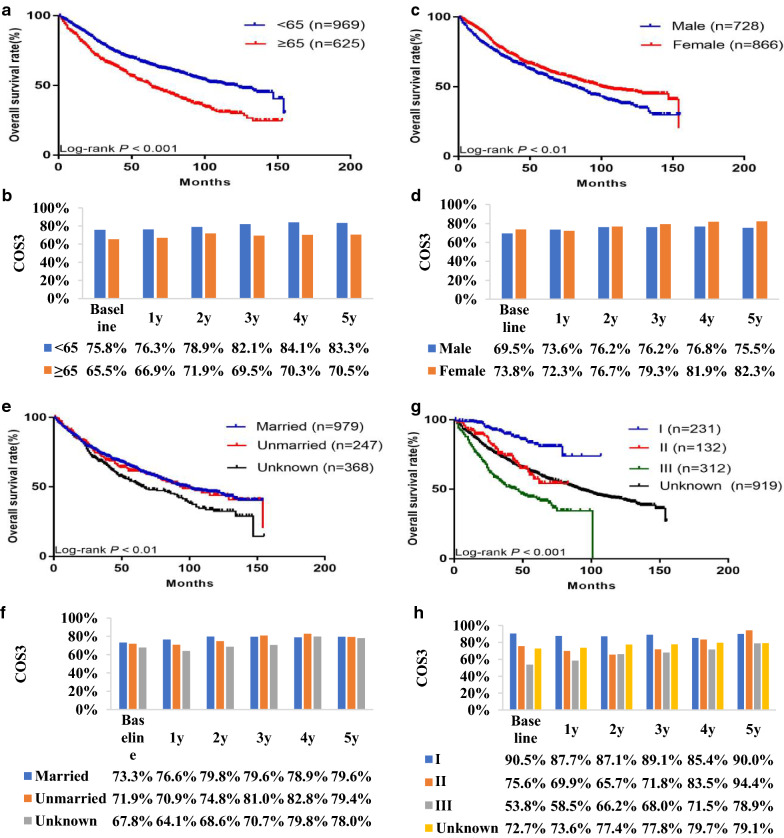
Actual overall survival stratified by: **a** age, **c** sex, **e** marital status, and **g** FNCLCC grade vs conditional overall survival relative to actual survival stratified by: **b** age, **d** sex, **f** marital status, and **h** ENETs NFNCLCC grade

**Fig. 5:**
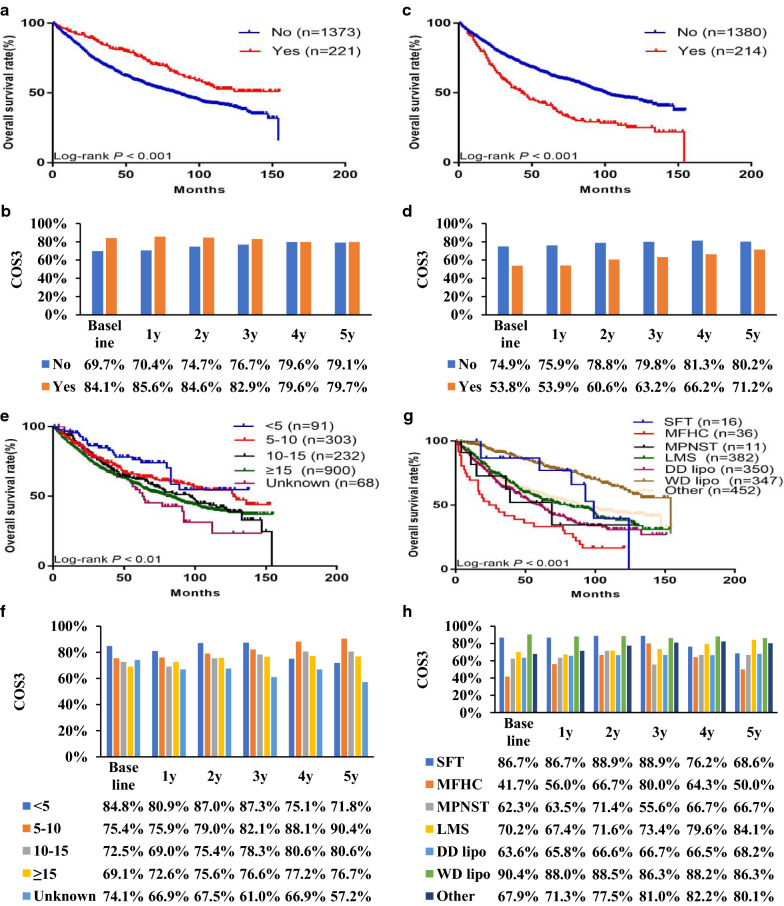
Actual overall survival stratified by: **a** multifocality, **c** chemotherapy, **e** size, and **g** histology vs conditional overall survival relative to actual survival stratified by: **b** multifocality, **d** chemotherapy, **f** size, and **h** histology

Subgroup analysis was performed to further evaluate the effect of independent prognostic factors on the OS and COS3 rates (Figs. 4 and 5). The OS rate decreased over time in each subgroup, while the COS3 rate had gradually increased after surgery. In addition, the COS3 rate exceeded the OS rate for each prognostic factor. Moreover, the difference between the OS and COS3 rates was more significant in patients with poor clinicopathological factors at baseline. In contrast, this difference was relatively small in patients with good initial clinicopathological factors at baseline. For example, among FNCLCC grade III patients, the 8-year OS rate was 34.3%, while the 5-year COS3 rate was 78.9% (Δ44.6%). In addition, among FNCLCC grade I patients, the 8-year OS rate was 74.0% and the 5-year COS3 rate was 90.0% (Δ16.0%). The gap between the COS3 rates was more significant in patients with poor clinicopathological factors at baseline. The COS3 rate of FNCLCC grade III patients increased by 20.4% (58.5–78.9%) within 5 years after surgery, while that of FNCLCC grade I patients increased by 2.3% (87.7–90.0%). The |*d*| value associated with the COS3 rate among subgroups, with the exception of the age subgroup, decreased over time. For example, the |*d*| value between grade I and grade III decreased from 0.82 at baseline to 0.51 at 3 years and then to 0.27 at 5 years (Table 3).

**Table 3:** Three-year conditional survival rates of patients with primary retroperitoneal tumors after surgical resection in relation to prognostic factors

Variable	COS3, %						CCSS3, %					
	Years since diagnosis						Years since diagnosis					
	Basline	1y	2y	3y	4y	5y	Basline	1y	2y	3y	4y	5y
Overall	71.8	72.9	76.5	77.9	79.6	79.3	77.1	78.1	81.3	82.7	84.9	85.8
*Age (year)*												
< 65	75.8	76.3	78.9	82.1	84.1	83.3	79.0	79.8	81.7	84.8	87.6	88.6
≥ 65	65.5	66.9	71.9	69.5	70.3	70.5	73.9	75.0	80.6	78.4	79.1	79.6
*d *(< 65 vs. ≥ 65)	0.23	0.21	0.17	0.31	0.35	0.31	0.12	0.12	0.03	0.16	0.24	0.26
*Sex*												
Male	69.5	73.6	76.2	76.2	76.8	75.5	75.1	79.5	81.7	81.7	82.1	83.9
Female	73.8	72.3	76.7	79.3	81.9	82.3	78.7	76.9	85.0	83.4	87.2	87.3
*d *(male vs. female)	− 0.10	0.03	− 0.01	− 0.08	− 0.13	− 0.17	− 0.09	0.06	− 0.08	− 0.04	− 0.14	− 0.10
*Marital status*												
Married	73.3	76.6	79.8	79.6	78.9	79.6						
Unmarried	71.9	70.9	74.8	81.0	82.8	79.4						
Unknown	67.8	64.1	68.6	70.7	79.8	78.0						
*d *(married vs. unmarried)	0.03	0.13	0.12	− 0.03	− 0.09	0.0						
*d *(married vs. unknown)	0.12	0.28	0.26	0.21	− 0.02	− 0.02	0.11	0.11	0.24	0.17	− 0.11	− 0.07
*FNCLCC grade*												
I	90.5	87.7	87.1	89.1	85.4	90.0	93.0	93.4	92.9	94.4	94.4	98.2
II	75.6	69.9	65.7	71.8	83.5	94.4	82.8	74.7	69.4	76.0	88.4	100.0
III	53.8	58.5	66.2	68.0	71.5	78.9	61.2	65.8	72.0	72.0	74.1	81.7
Unknown	72.7	73.6	77.4	77.8	79.7	79.1	77.6	78.2	81.7	82.6	85.3	85.6
*d* (I vs. II)	0.33	0.40	0.50	0.42	0.05	− 0.11	0.24	0.46	0.60	0.49	0.17	− 0.05
*d* (I vs. III)	0.82	0.65	0.50	0.51	0.35	0.27	0.76	0.67	0.54	0.59	0.56	0.47
*d* (I vs. Unknown)	0.40	0.32	0.23	0.27	0.14	0.66	0.37	0.37	0.29	0.31	0.25	0.36
*Size (cm)*												
< 5	84.8	80.9	87.0	87.3	75.1	71.8	90.9	86.4	91.7	89.1	75.1	71.8
5–10	75.4	75.9	79.0	82.1	88.1	90.4	80.6	80.8	82.9	85.5	89.5	92.4
10–15	72.5	69.0	75.4	78.3	80.6	80.6	78.6	75.5	80.5	82.9	86.4	87.3
≥ 15	69.0	72.6	75.6	76.6	77.2	76.7	74	77.3	80.5	81.5	83.5	84.6
Unknown	74.1	66.9	67.5	61.0	66.9	57.2	79.5	73.8	74.6	74.1	83.7	74.8
*d* (< 5 vs. 5–10)	0.21	0.11	0.19	0.13	− 0.33	− 0.45	0.25	0.14	0.23	0.09	− 0.40	− 0.59
*d* (< 5 vs. 10–15)	0.27	0.26	0.28	0.22	0.14	− 0.21	0.29	0.27	0.29	0.16	− 0.31	− 0.44
*d* (< 5 vs. ≥ 15)	0.35	0.18	0.27	0.26	0.05	− 0.12	0.40	0.22	0.29	0.20	− 0.23	− 0.37
*d* (< 5 vs. Unknown)	0.24	0.32	0.46	0.63	0.20	0.89	0.27	0.30	0.44	0.40	− 0.24	− 0.09
*Multifocality*												
No	69.7	70.4	74.7	76.7	79.6	79.1	73.6	74.2	77.8	79.5	87.1	83.1
Yes	84.1	85.6	84.6	82.9	79.6	79.7	97.5	98.4	97.5	95.9	94.7	96.0
*d* (No vs. Yes)	− 0.32	− 0.34	− 0.24	− 0.15	0	− 0.01	− 0.57	− 0.59	− 0.51	− 0.43	− 0.21	− 0.37
*Histology*												
SFT	86.7	86.7	88.9	88.9	76.2	68.6	86.7	86.7	88.9	88.9	76.2	68.6
MFHC	41.7	56.0	66.7	80.0	64.3	50.0	49.2	62.0	70.6	80	71.4	64.8
MPNST	62.3	63.5	71.4	55.6	66.7	66.7	76.2	76.2	85.7	100.0	100.0	100.0
LMS	70.2	67.4	71.6	73.4	79.6	84.1	75.6	72.6	75.6	75.7	81.6	85.7
DD lipo	63.6	65.8	66.6	66.7	66.5	68.2	68.7	70.7	72.4	72.3	74.2	76.9
WD lipo	90.4	88.0	88.5	86.3	88.2	86.3	95.6	93.8	94.2	91.7	92.4	91.7
Other	67.9	71.3	77.5	81.0	82.2	80.1	72.7	76.1	81.5	86.4	88.9	88.7
*d* (SFT vs. MFHC)	1.00	0.68	0.53	0.22	0.30	0.45	0.89	0.60	0.47	0.23	0.13	0.11
*d* (SFT vs. MPNST)	0.54	0.52	0.42	0.81	0.24	0.05	0.25	0.26	0.08	− 0.29	− 0.66	− 0.90
*d* (SFT vs. LMS)	0.37	0.43	0.41	0.38	− 0.09	− 0.38	0.26	0.34	0.34	0.35	− 0.15	− 0.49
*d* (SFT vs. DD lipo)	0.51	0.46	0.53	0.54	0.24	0.01	0.43	0.39	0.42	0.44	0.06	− 0.24
*d* (SFT vs. WD lipo)	− 0.08	− 0.03	0.01	0.06	− 0.30	− 0.43	− 0.21	− 0.17	− 0.14	− 0.07	− 0.45	− 0.66
*d *(SFT vs. Other)	0.42	0.34	0.27	0.19	− 0.15	− 0.28	0.33	0.26	0.19	0.07	− 0.35	− 0.57
*Chemotherapy*												
No	74.9	75.9	78.8	79.8	81.3	80.2	80.6	81.3	83.8	84.5	86.5	86.7
Yes	53.8	53.9	60.6	63.2	66.2	71.2	56.2	58.0	64.1	68.6	72.2	77.7
*d *(No vs. Yes)	0.47	0.49	0.43	0.40	0.38	0.22	0.58	0.57	0.51	0.42	0.40	0.26

*SFT* solitary fibrous tumor, *MFHC* Malignant fibrous histiocytoma, *MPNST* malignant peripheral nerve sheath tumor, *LMS* leiomyosarcoma, *DD lipo* dedifferentiated liposarcoma, *WD lipo* well-differentiated liposarcoma, *FNCLCC* French National Federation of the Centers for the Fight Against Cancer, *API* Asian/Pacific Islander, *COS3* 3-year conditional overall survival, *CCSS3* 3-year conditional cancer-specific survival, *y* year, *d* standardized difference

### CCSS and comparison with actual CSS

The actual CSS and CCSS3 rates after 3 years are shown in Fig. 3b. The postoperative CCSS3 rates increased yearly, while the actual CSS rates decreased. The CCSS3 rates increased by 1.0 (78.1%), 5.6 (82.7%), 8.7 (85.8%) at 1, 3, and 5 years, respectively, while the corresponding actual CSS rates decreased by 5.3 (71.8%), 13.4 (63.7%), and 18.9 (58.2%) at 4, 6, and 8 years, respectively.

The detailed CCSS3 rates of patients who survived to specific time points are shown in Additional file 3: Table S3. For example, among patients surviving at 1, 2, 3, and 4 years after surgery, the probability of not dying due to cancer at year 5 would be 73.8, 81.3, 88.0, and 94.5%, respectively.

Subgroup analysis of the independent prognostic factors associated with CSS was performed to further evaluate the impact of clinicopathological features on the CSS and CCSS3 rates (Additional file 4: Figures S1 and 2). The CSS rate of each subgroup decreased with time, while the CCSS3 rate gradually increased after surgery. In addition, for each prognostic factor, the CCSS3 rate exceeded the CSS rate. Moreover, the differences between the CSS and CCSS3 rates were more significant in patients with poor clinicopathological factors. On the contrary, among patients with good clinicopathological factors, the differences between the CSS and CCSS3 rates were relatively small. For example, among FNCLCC grade III patients, the CSS rate at 8 years was 41.3% and the CCSS3 rate at 5 years was 81.7% (Δ40.4%), while among FNCLCC grade I patients, the CSS rate at 8 years was 87.8% and the CCSS3 rate at 5 years was 98.2% (Δ10.4%). Notably, the gap between the CCSS3 rates was more significant in patients with poor clinicopathological factors at baseline. The CCSS3 rates of FNCLCC grade III patients increased by 15.9% (65.8–81.7%) within 5 years after surgery, while those of FNCLCC grade I patients increased by 4.8% (93.4–98.2%). The |*d*| value associated with the CCSS3 rate among subgroups, with the exception of the age subgroup, had decreased over time. For example, the |*d*| value between chemotherapy and no chemotherapy decreased from 0.58 at baseline to 0.42 at 3 years and then to 0.26 at 5 years (Table 3).

## Discussion

A primary RPT is commonly malignant with a very complex pathology. Early diagnosis and treatment tend to be difficult because most patients are resistant to chemotherapy and radiotherapy, the tumor volume is often larger, and the tumor is usually accompanied by lymphovascular, perineural, and organ invasion. Thus, the prognosis of these patients is often poor [29, 30]. In fact, the 7-year OS rates of patients with RPTs with different pathological factors range between 30 and 50% [31]. However, the impacts of pathological factors on patient prognosis remain unclear.

In the present study, age, sex, FNCLCC grade, multifocality, histology, and chemotherapy were identified as independently predictive of OS and CSS (all, *p* < 0.05). Moreover, age ≥ 65 years was correlated with a poorer prognosis. Likewise, Gronchi et al. [10] found that advanced age was associated with a poorer prognosis [hazard ratio (HR) = 1.34; 95% confidence interval (CI) = 1.04–1.21; *p* < 0.05]. As a possible explanation for this finding, perception and response decrease with age, resulting in later onset of symptoms, and the elderly are often complicated by systemic pulmonary and circulatory diseases prior to surgery, which results in higher incidences of postoperative complications and poor prognoses. Toulmonde et al. [32] reported that the prognosis of male patients with retroperitoneal sarcoma was poor (HR = 1.7; 95% CI = 1.3–2.3; *p* < 0.001), which is consistent with our results, suggesting that estrogen therapy may be beneficial to RPT patients, similar to those with colorectal cancer [33]. Moreover, Stoeckle et al. [34] found that the probability of metastasis of grade 3 disease was 24.6%, while that of grade 2 was 24.6%, and that of grade 1 was 0. The probability of complete resection of grade 3 disease was 44%, that of grade 2 was 38%, and that of grade 1 was 11%. Moreover, the prognosis of patients with grade 3 disease is exceptionally poor (HR = 3.39; 95% CI = 1.51–7.61; *p* < 0.01). In general, the higher the grade, the more likely metastasis. In this scenario, surgery becomes more difficult, resulting in poor prognoses, consistent with the results of a previous study [34]. In the present study, the prognosis of patients with a tumor ≥ 15 cm was poor. Stoeckle et al. [34] found that tumors of > 10 vs. < 5 cm were associated with a higher rate of distant metastasis (15.9 vs. 12.5%, respectively), while a tumor > 10 vs. < 10 cm was associated with a lower rate of complete resection (71 vs. 60%, respectively, *p* < 0.001). Gronchi et al. [10] stated that the larger the tumor volume, the higher the patient's risk score and, thus, a poorer prognosis. Luo et al. [35] found that tumor size ≥ 10 cm was an independent prognostic factor for patients with a solitary primary retroperitoneal fibrous sarcoma (HR = 6.03; 95% CI = 1.18–30.77; *p* = 0.031). Multifocal RPTs are more likely to metastasize, resulting in a poorer prognosis than that of a single RPT (HR = 2.40; 95% CI = 1.44–4.02; *p* < 0.001) [10], which is consistent with our results. In the present study, only MFHC had a significantly worse prognosis than a borderline solitary fibrous tumor. However, the influence of the pathological type on prognosis remains controversial. Gronchi et al. [10] found that a well-differentiated liposarcoma (WD lipo) has the highest risk score, as determined by nomography, and the prognosis is worse than that of a dedifferentiated liposarcoma (DD lipo). Some scholars believe that as compared with a WD lipo, a DD lipo and LMS are more likely to metastasize to distant tissues, resulting in a poorer prognosis [32]. The incidence of distant metastasis of MFHC is reportedly 12%, while that of liposarcoma was not observed [34], indicating that the prognosis of MFHC may be worse than that of liposarcoma.

To date, no randomized controlled trial has yet to compare the efficacy of adjuvant therapy vs. surgical resection for RPTs, and there is no evidence that adjuvant chemotherapy is beneficial to patients following complete resection of a RPT [36, 37]. In the present study, chemotherapy offered no survival benefit to patients, as the side effects outweigh the benefits. However, chemotherapy combined with hyperthermia, extracorporeal radiotherapy, or chemoradiotherapy was safe with no fatal complications for carefully selected patients with RPTs. For chemotherapy-sensitive tumors, such as synovial sarcoma and LMS, radiotherapy is recommended for solitary fibrous tumors [38–45].

Conventional prognostic assessment of patients with primary RPTs is usually based on the pathological stage, type, and grade, and cumulative survival is calculated based on follow-up data after diagnosis. This traditional survival assessment approach provides a static risk assessment, but does not take into account changes in risks associated with postoperative survival. However, the risk of death after diagnosis is not constant, but changes with prolonged survival [46]. As compared with traditional assessment methods, CS has the advantage of reflecting the survival probability that changes over time, which may be more useful for prediction of prognosis.

Several conclusions can be drawn from the results of the present study. First, unlike traditional OS, CSS showed a downward trend over time, while COS3 and CCSS3 showed an upward trend over time. In particular, the CCSS3 rate increased to more than 80% at 2 years after surgery, indicating that these patients had a higher expectation of cancer-free survival. With the extension of survival time, the COS3 and CCSS3 rates were higher than the actual survival rate at each time point. Additionally, patients with an initial poor prognosis had a greater gap between the actual survival rate and estimated COS3 and CCSS3 rates, which tended to significantly increase over time. Hence, this dynamic prediction can help to reduce patient anxiety and bestow confidence in long-term survival, especially for patients with pre-existing poor pathological factors. In addition, the |*d*| values between prognostic factors, in addition to age, gradually decreased over time, while the gap between groups became smaller, suggesting that some high risk patients may die soon after surgery, while the prognoses of surviving patients with high risk factors will be close to those of patients with some low risk factors as time goes on, which can also reduce anxiety and improve quality of life, especially for high-risk patients [47].

In addition, our research provided certain guiding principles for follow-up strategies. As time progressed, the risk of death decreased, and CS may reached a threshold value. For example, the COS3 rate of patients aged < 65 years increased to 82.1% at year 3, while the COS3 rate of those aged ≥ 65 years did not reach 80% in year 5, and the COS3 rate of patients with grade I disease reached 80% after surgery, suggesting that a 5-year follow-up may be insufficient for patients with high risk factors and may be too long for those with a better prognosis. This conclusion can help formulate individualized follow-up strategies, reduce unnecessary follow-ups for patients, and save medical costs, while reducing the anxiety of patients due to the fear of tumor recurrence and improving quality of life.

Because it is very difficult to collect a large number of patients with low morbidity, there were some limitations to this study that should be addressed. First, this was a retrospective study, thus there was a certain degree of selection bias. Because neoadjuvant therapy may have a down-staging effect and affect the impact of various prognostic factors, patients receiving neoadjuvant therapy were excluded from analysis. Undeniably, neoadjuvant chemotherapy has a certain impact on patient prognosis. Second, because the SEER database lacked information on Asian and European patients, the universality of our conclusions was reduced. Nonetheless, this study is the first to include recent large sample data for clinical analysis and fully included various clinicopathological factors that may affect OS and CSS, which is of great significance for dynamic prognosis assessment of patients, while enabling accurate and individualized follow-up strategies.

## Conclusions

Finally, in this study, age, sex, FNCLCC grade, multifocality, histology, and chemotherapy were identified as independent prognostic factors of OS and CSS. The postoperative CS of primary RPTs was dynamic and increased with time, especially for patients with poor clinicopathological characteristics at baseline. Therefore, CS can provide more valuable and accurate evaluations of the long-term prognoses of patients with primary RPTs.

## Supplementary information

**Additional file 1: Table S1.** Baseline characteristics of the study population.

**Additional file 2: Table S2.** The univariate analyses of factors associated with overall survival and cancer-specific survival.

**Additional file 3: Table S3.** The probability that patients with primary retroperitoneal tumors after surgical resection will remain alive at a specific time point given that they have already survived for a certain amount of time.

**Additional file 4: Figure S1.** Actual cancer-specific survival stratified by: (A) age, (C) sex, (E) FNCLCC grade, and (G) size vs conditional cancer-specific survival relative to actual survival stratified by: (B) age, (D) sex, (F) FNCLCC grade, and (H) size. **Figure S2.** Actual cancer-specific survival stratified by: (A) multifocality, (C) chemotherapy, and (E) histology vs conditional cancer-specific survival relative to actual survival stratified by: (B) multifocality, (D) chemotherapy, (F) histology.

## Data Availability

Not applicable.
