# Supplementary material for: Conditional survival after surgical resection of primary retroperitoneal tumors: a population-based study
Source: Cancer Cell Int. 2021 Jan 20;21:60. doi: 10.1186/s12935-021-01751-z (PMC7816497; doi:10.1186/s12935-021-01751-z)
Supplement: Supplementary file 1 — Additional file 1: Table S1. Baseline characteristics of the study population. [file 12935_2021_1751_MOESM1_ESM.docx]

TableS1 Baseline characteristics of the study population

| Variable |  |
| --- | --- |
|  | Total [n (%)] |
| Age (year) | 1594 |
| <65 | 969 (60.8) |
| ≥65 | 625 (39.2) |
| Sex |  |
| Male | 728 (45.7) |
| Female | 866 (54.3) |
| Race |  |
| White | 1267 (79.5) |
| Black | 152 (9.5) |
| API | 152 (9.5) |
| Other | 23 (1.5) |
| Marital status |  |
| Married | 979 (61.4) |
| Unmarried | 247 (15.5) |
| Unknown | 368 (23.1) |
| FNCLCC grade |  |
| Ⅰ | 231 (14.5) |
| Ⅱ | 132 (8.3) |
| Ⅲ | 312 (19.6) |
| Unknown | 919 (57.6) |
| Size (cm) |  |
| <5 | 91 (5.7) |
| 5-10 | 303 (19.0) |
| 10-15 | 232 (14.6) |
| ≥15 | 900 (56.5) |
| Unknown | 68 (4.2) |
| Multifocality |  |
| No | 1373 (86.1) |
| Yes | 221 (13.9) |
| Histology |  |
| SFT | 16 (1.0) |
| MFHC | 36 (2.3) |
| MPNST | 11 (0.7) |
| LMS | 382 (24.0) |
| DD lipo | 350 (22.0) |
| WD lipo | 347 (21.8) |
| Other | 452 (28.2) |
| Radiation |  |
| No | 1201 (75.3) |
| Yes | 393 (24.7) |
| Chemotherapy |  |
| No | 1380 (86.6) |
| Yes | 214 (13.4) |
| Chemoradiotherapy |  |
| No | 1520 (95.4) |
| Yes | 74 (4.6) |
| Extent of resection |  |
| Complete | 840 (52.7) |
| Incomplete | 721 (45.2) |
| Unknown | 33 (2.1) |

SFT, solitary fibrous tumor; MFHC, Malignant fibrous histiocytoma; MPNST, malignant peripheral nerve sheath tumor; LMS, leiomyosarcoma; DD lipo, dedifferentiated liposarcoma; WD lipo, well-differentiated liposarcoma; FNCLCC, French National Federation of the Centers for the Fight Against Cancer; API, Asian/Pacific Islander
