# Supplementary material for: Conditional survival after surgical resection of primary retroperitoneal tumors: a population-based study
Source: Cancer Cell Int. 2021 Jan 20;21:60. doi: 10.1186/s12935-021-01751-z (PMC7816497; doi:10.1186/s12935-021-01751-z)
Supplement: Supplementary file 2 — Additional file 2: Table S2. The univariate analyses of factors associated with overall survival and cancer-specific survival. [file 12935_2021_1751_MOESM2_ESM.docx]

Table S2 The univariate analyses of factors associated with overall survival and cancer-specific survival

| Variable | OS-univariate Cox  regression | | CSS- univariate Cox  regression | |
| --- | --- | --- | --- | --- |
|  | HR (95% CI) | P-value | HR (95% CI) | P-value |
| Age |  |  |  |  |
| <65 | 1 |  | 1 |  |
| ≥65 | 1.715 (1.476-1.992) | <0.001 | 1.394 (1.171-1.659) | <0.001 |
| Sex |  |  |  |  |
| Male | 1 |  | 1 |  |
| Female | 0.800(0.689-0.928) | 0.003 | 0.820(0.691-0.974) | 0.024 |
| Race |  |  |  |  |
| White | 1 |  | 1 |  |
| Black | 0.917(0.705-1.192) | 0.516 | 1.006(0.752-1.346) | 0.969 |
| API | 0.830(0.635-1.085) | 0.173 | 0.887(0.656-1.199) | 0.434 |
| Other | 0.850(0.423-1.707) | 0.647 | 0.567(0.212-1.519) | 0.259 |
| Marital status |  |  |  |  |
| Married | 1 |  | 1 |  |
| Unmarried | 1.027(0.825-1.279) | 0.809 | 1.141(0.896-1.453) | 0.285 |
| Unknown | 1.351(1.135-1.608) | <0.001 | 1.278(1.042-1.568) | 0.018 |
| FNCLCC grade |  |  |  |  |
| Ⅰ | 1 |  | 1 |  |
| Ⅱ | 2.533(1.576-4.071) | <0.001 | 3.318(1.843-5.976) | <0.001 |
| Ⅲ | 4.729(3.213-6.959) | <0.001 | 6.643(4.052-10.891) | <0.001 |
| Unknown | 2.713(1.881-3.913) | <0.001 | 3.653(2.269-5.880) | <0.001 |
| Size (cm) |  |  |  |  |
| <5 | 1 |  | 1 |  |
| 5-10 | 1.251(0.817-1.914) | 0.303 | 1.312(0.798-2.155) | 0.284 |
| 10-15 | 1.517(0.987-2.329) | 0.057 | 1.561(0.946-2.577) | 0.081 |
| ≥15 | 1.644(1.106-2.443) | 0.014 | 1.734(1.091-2.755) | 0.020 |
| Unknown | 1.926(1.163-3.190) | 0.011 | 1.752(0.960-3.198) | 0.068 |
| Multifocality |  |  |  |  |
| No | 1 |  | 1 |  |
| Yes | 0.582(0.460-0.736) | <0.001 | 0.107(0.060-0.190) | <0.001 |
| Histology |  |  |  |  |
| SFT | 1 |  | 1 |  |
| MFHC | 2.748(1.207-6.257) | 0.016 | 2.548(1.041-6.234) | 0.040 |
| MPNST | 1.620 (0.544-4.823) | 0.386 | 0.616 (0.124-3.054) | 0.553 |
| LMS | 1.324(0.622-2.818) | 0.466 | 1.249(0.552-2.825) | 0.594 |
| DD lipo | 1.624(0.763-3.457) | 0.208 | 1.475(0.652-3.340) | 0.351 |
| WD lipo | 0.530(0.244-1.149) | 0.108 | 0.303(0.128-0.717) | 0.007 |
| Other | 1.243(0.585-2.640) | 0.571 | 1.110(0.491-2.508) | 0.802 |
| Radiation |  |  |  |  |
| No | 1 |  | 1 |  |
| Yes | 0.931(0.785-1.104) | 0.410 | 0.999(0.824-1.212) | 0.992 |
| Chemotherapy |  |  |  |  |
| No |  |  |  |  |
| Yes | 1.800(1.492-2.171) | <0.001 | 2.203(1.798-2.699) | <0.001 |
| Chemoradiotherapy |  |  |  |  |
| No | 1 |  | 1 |  |
| Yes | 1.396(1.038-1.878) | 0.027 | 1.591(1.152-2.197) | 0.005 |
| Extent of resection |  |  |  |  |
| Complete | 1 |  | 1 |  |
| Incomplete | 0.962(0.827-1.119) | 0.611 | 0.855(0.718-1.019) | 0.080 |
| Unknown | 1.106(0.660-1.854) | 0.703 | 1.010(0.553-1.845) | 0.973 |

SFT, solitary fibrous tumor; MFHC, Malignant fibrous histiocytoma; MPNST, malignant peripheral nerve sheath tumor; LMS, leiomyosarcoma; DD lipo, dedifferentiated liposarcoma; WD lipo, well-differentiated liposarcoma; FNCLCC, French National Federation of the Centers for the Fight Against Cancer; API, Asian/Pacific Islander; OS, overall survival; CSS, cancer-specific survival
