# Supplementary material for: Conditional survival after surgical resection of primary retroperitoneal tumors: a population-based study
Source: Cancer Cell Int. 2021 Jan 20;21:60. doi: 10.1186/s12935-021-01751-z (PMC7816497; doi:10.1186/s12935-021-01751-z)
Supplement: Supplementary file 3 — Additional file 3: Table S3. The probability that patients with primary retroperitoneal tumors after surgical resection will remain alive at a specific time point given that they have already survived for a certain amount of time. [file 12935_2021_1751_MOESM3_ESM.docx]

Table S3 The probability that patients with primary retroperitoneal tumors after surgical resection will remain alive at a specific time point given that they have already survived for a certain amount of time

| Total cancer-specific survival time, y | If the patient has survived, % | | | | | | | |
| --- | --- | --- | --- | --- | --- | --- | --- | --- |
|  | 1y | 2y | 3y | 4y | 5y | 6y | 7y | 8y |
| 1 | 100.0 |  |  |  |  |  |  |  |
| 2 | 90.7 | 100.0 |  |  |  |  |  |  |
| 3 | 83.8 | 92.3 | 100.0 |  |  |  |  |  |
| 4 | 78.1 | 86.0 | 93.1 | 100.0 |  |  |  |  |
| 5 | 73.8 | 81.3 | 88.0 | 94.5 | 100.0 |  |  |  |
| 6 | 69.3 | 76.4 | 82.7 | 88.8 | 93.9 | 100.0 |  |  |
| 7 | 66.3 | 73.1 | 79.1 | 84.9 | 89.9 | 95.7 | 100.0 |  |
| 8 | 63.3 | 69.8 | 75.5 | 81.1 | 85.8 | 91.3 | 95.5 | 100.0 |
