# Supplementary figures and images for: Conditional survival after surgical resection of primary retroperitoneal tumors: a population-based study
Source: Cancer Cell Int. 2021 Jan 20;21:60. doi: 10.1186/s12935-021-01751-z (PMC7816497; doi:10.1186/s12935-021-01751-z)

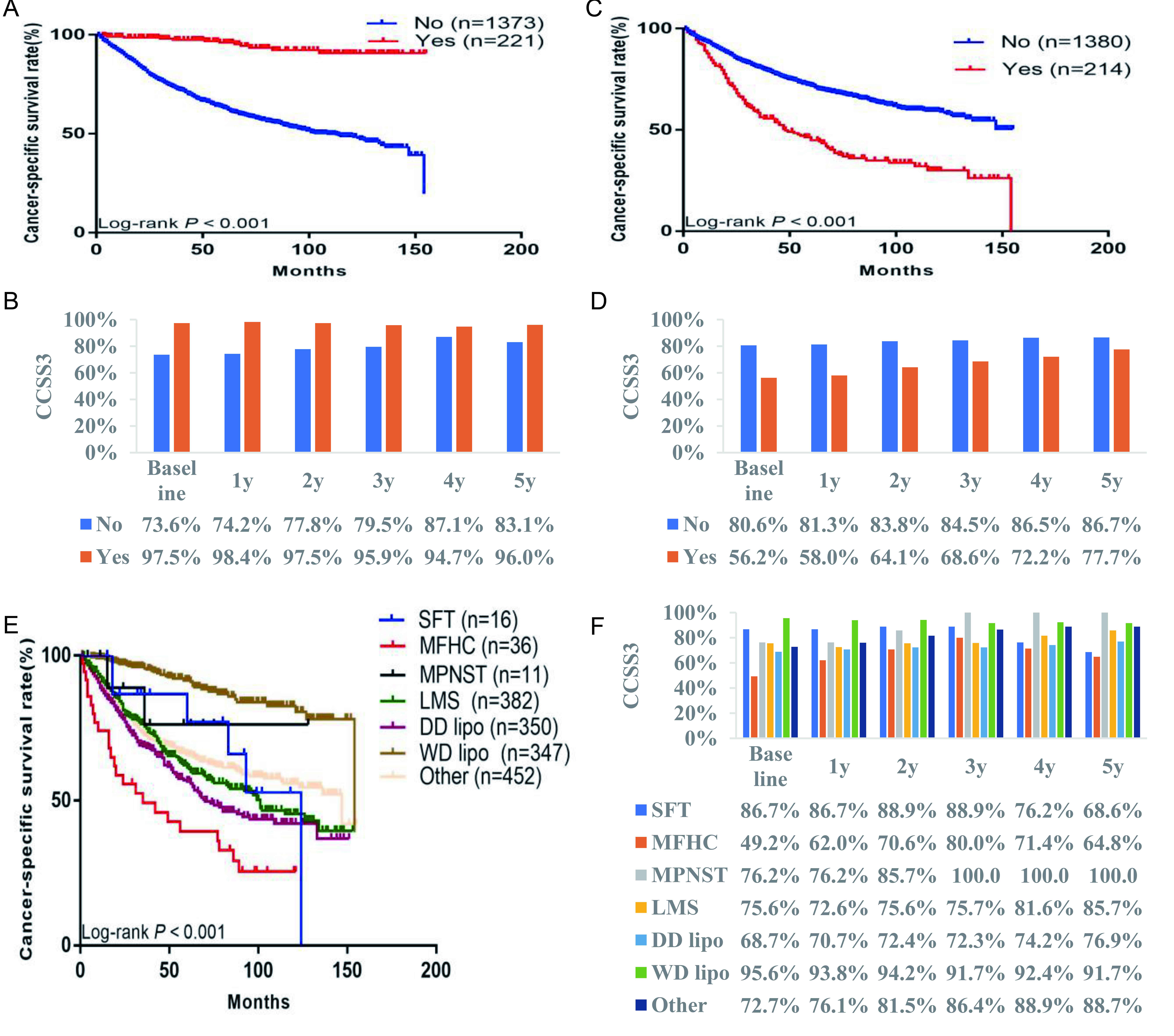

Supplement: Supplementary file 4 — Additional file 4: Figure S1. Actual cancer-specific survival stratified by: (A) age, (C) sex, (E) FNCLCC grade, and (G) size vs conditional cancer-specific survival relative to actual survival stratified by: (B) age, (D) sex, (F) FNCLCC grade, and (H) size. Figure S2. Actual cancer-specific survival stratified by: (A) multifocality, (C) chemotherapy, and (E) histology vs conditional cancer-specific survival relative to actual survival stratified by: (B) multifocality, (D) chemotherapy, (F) histology. [file 12935_2021_1751_MOESM4_ESM.docx]
